# Why Acting Environmentally-Friendly Feels Good: Exploring the Role of Self-Image

**DOI:** 10.3389/fpsyg.2016.01846

**Published:** 2016-11-24

**Authors:** Leonie A. Venhoeven, Jan Willem Bolderdijk, Linda Steg

**Affiliations:** ^1^Department of Psychology, University of GroningenGroningen, Netherlands; ^2^Department of Marketing, University of GroningenGroningen, Netherlands

**Keywords:** environmentally friendly behavior, pro-environmental behavior, self-image, positive emotions, well-being, autonomy

## Abstract

Recent research suggests that engagement in environmentally-friendly behavior can feel good. Current explanations for such a link do not focus on the nature of environmentally-friendly behavior itself, but rather propose well-being is more or less a side-benefit; behaviors that benefit environmental quality (e.g., spending one's money on people rather than products) also tend to make us feel good. We propose that the moral nature of environmentally-friendly behavior itself may elicit positive emotions as well, because engaging in this behavior can signal one is an environmentally-friendly and thus a good person. Our results show that engagement in environmentally-friendly behavior can indeed affect how people see themselves: participants saw themselves as being more environmentally-friendly when they engaged in more environmentally-friendly behavior (Study 1). Furthermore, environmentally-friendly behavior resulted in a more positive self-image, more strongly when it was voluntarily engaged in, compared to when it was driven by situational constraints (Study 2). In turn, the more environmentally-friendly (Study 1) and positive (Study 2) people saw themselves, the better they felt about acting environmentally-friendly. Together, these results suggest that the specific self-signal that ensues from engaging in environmentally-friendly behavior can explain why environmentally-friendly actions may elicit a good feeling.

## Introduction

Increasing environmental quality is an important goal for many governments around the world. As for instance agreed upon during the Paris climate conference (COP21), it is an international aim to keep global temperature rises well below 2°C above pre-industrial levels (European Commission, December 23, 2015). At the same time, an increasing number of governments see citizens' well-being as an important indicator of a country's welfare. As a result, well-being research is more frequently used as a guide to develop policies that enable people to live better lives (Helliwell et al., 2012).

Yet, striving toward a better environmental quality and higher human well-being are sometimes seen as separate, possibly even conflicting goals, as acting environmentally-friendly often involves some degree of effort and discomfort (De Young, 1990-1991; Lorenzoni et al., 2007). In the current studies, we wonder whether reaching environmental quality and human well-being are necessarily at odds. Opposite to the negative view of environmentally-friendly behavior, research shows that people who act environmentally-friendly experience more happiness and higher life satisfaction (Kasser and Sheldon, 2002; Brown and Kasser, 2005; Xiao and Li, 2011). The reason why such a positive relationship between environmentally-friendly behavior and well-being may exist, however, remains unclear (Venhoeven et al., 2013). To answer this question, we will examine *why* engaging in environmentally-friendly behavior may actually contribute to individual well-being. We do so by examining the impact of environmentally-friendly actions on one common operationalization of well-being—positive emotions.

Explanations given for a link between this type of behavior and feeling good often do not focus on the nature of environmentally-friendly behavior itself, but rather propose well-being is more or less a side-benefit; behaviors that benefit environmental quality also happen to make us feel good. Some suggest that the things that actually make people happy, like social relationships and personal growth, can be achieved in sustainable ways. They are often contrasted to (over)consumption and materialism, which are linked to unsustainable behavior and are found to have a less strong positive or even a negative effect on happiness. By focusing on those things that actually make them happy, instead of focusing on consumption and materialism, people can thus live more sustainably, and feel better at the same time (Csikszentmihalyi, 2000; Jackson, 2005; Beavan, 2009; Kasser, 2009). Others propose that specific personal traits such as being mindful both make people act environmentally-friendly and increase a good feeling (Brown and Kasser, 2005). We wonder whether a link between environmentally-friendly behavior and positive emotions are indeed mainly a side-benefit. Could the nature of environmentally-friendly behavior *itself* not make people feel good as well?

In the current studies, we focus on the role one's self-image may play in explaining the relationship between environmentally-friendly behavior and positive emotions. More specifically, we suggest acting environmentally-friendly itself may feel good because this behavior can signal something positive about who you are.

Because of the positive consequences environmentally-friendly behavior has for nature and other people now and in the future, acting this way can be seen as moral behavior (Leopold, 1949; Heberlein, 1972; Thøgersen, 1996). As research shows, many people agree that nature has intrinsic value, and humans have moral duties and obligations to animals, plants, and non-living nature (Leiserowitz et al., 2005), and a moral responsibility to address climate change (Lorenzoni et al., 2007). The choice of engaging in environmentally-friendly behavior is thus based, amongst others, on wanting to do the moral thing (Schwartz, 1977; Schwartz and Howard, 1981; Lindenberg and Steg, 2007).

One of the pillars on which people base their self-image, is their own actions (Bem, 1967, 1972). As Bem (1972) proposes “Individuals come to “know” their own attitudes, emotions, and other internal states partially by inferring them from observations of their own overt behavior and/or the circumstances in which this behavior occurs” (p. 2). How moral people perceive their behavior to be may thereby affect one's moral self-image, a central part of the more overall positive self-concept (Aquino and Reed, 2002; Dunning, 2007; Sachdeva et al., 2009). When they engage in morally good behavior, people see themselves as a moral person and conclude they must be a good person as well.

Research shows that engagement in environmentally-friendly behavior can indeed influence how people see themselves. Acting environmentally-friendly can lead to a more environmentally-friendly self-identity (Cornelissen et al., 2008; Van der Werff et al., 2014b) in that when people behave environmentally-friendly, they tend to see themselves more strongly as environmentally-friendly persons. If environmentally-friendly behavior is perceived to be a manifestation of morality, as we reason above, engagement in this behavior may furthermore elicit an overall positive self-image. Indeed, environmentally-friendly behavior has led people to see themselves in a more general positive light as well (Taufik et al., 2015). Additionally, how positively people think of themselves is an important determinant of how good they feel (Taylor and Brown, 1988; Baumeister, 1993). If perceiving one's actions to be environmentally-friendly leads to a positive self-image, this self-image may thus in turn elicit positive emotions.

As the quote by Bem (1972) above already illustrates, the circumstances in which behavior occurs can affect how the behavior itself is interpreted. In the current studies, we look specifically at the role of volition in this process. An important stance in theoretical considerations about morality is that “decisions are classified as moral only when the person who makes them is perceived to be the responsible agent, that is, to have chosen the action knowingly and willingly when he could have done otherwise” (p. 81, Heberlein, 1972). Following this reasoning, the same environmentally-friendly action will particularly be interpreted as a morally good behavior when the person actively chose (rather than was forced to) pursue it. Furthermore, when people voluntarily choose to engage in certain behavior, they are more likely to attribute the choice for engagement to internal instead of external causes (Ryan and Deci, 2000a,b; Van der Werff et al., 2014b). In sum, *making the choice* to engage in certain behavior rather than acting out of situational constraints may particularly reveal something about who you are—not only to others, but also to yourself (Bodner and Prelec, 2003). Following this reasoning, we wonder whether everyone who acts in an environmentally-friendly way will feel good about their engagement. If the self-signal this behavior sends is an important reason why acting environmentally-friendly feels good, especially people who behave this way out of their own volition, rather than out of situational constraints, should experience positive emotions about their behavior.

## The current studies

In the current studies, we aim to test whether acting environmentally-friendly feels good because this behavior signals something positive about who you are, and whether this effect is stronger when the choice for the behavior is made voluntarily.

We expect that acting environmentally-friendly feels good because it positively affects people's self-image. Following our reasoning above, we included two indicators of self-image: environmental self-identity and general positive self-image. More specifically, we expect people will see themselves as more environmentally-friendly (Study 1) and experience a boost in their more general positive self-image (Study 2) when they perceive their behavior to be more environmentally-friendly. We expect these effects to be more pronounced when engagement in this behavior is voluntary. In turn, we expect that the more environmentally-friendly and the more positive people's self-image is, respectively, the better they will feel about engaging in this behavior. Following this reasoning, we expect that people will feel better about more environmentally-friendly and voluntarily chosen behavior, and that this relationship is mediated by people's self-image.

## Study 1

### Method

We approached participants in a Dutch supermarket right after they paid for their groceries, asking them whether they had time to complete a short survey about the products they just bought. No incentives were provided for completion. In total, 178 people (80 female, 90 male, 8 unknown; *M*_*age*_ = 31.6 years, *SD*_*age*_ = 14.8; 54.7% of the sample had a monthly income of €500–€1000 or less; 76.4% of the sample had at least higher vocational education) agreed to complete the questionnaire that took approximately 10 min to fill out. In total 18 participants were excluded from the analyses because they had missing variables on at least one of the variables included in the analyses. The Ethical Committee Psychology of the University of Groningen approved Study 1 (approval number ppo-013-260). Informed consent was obtained from all participants.

How environmentally-friendly participants perceived their purchase to be, our independent variable, was operationalized in two ways. First, participants indicated whether they just bought any environmentally-friendly products (yes/no). The label “environmentally-friendly” was not defined, to allow participants to include any products they personally deemed environmentally-friendly. Although, this may mean they included products that are not actually environmentally-friendly in their count, we assume that it is people's *perception* of environmentally-friendliness, and not the actual environmentally-friendliness of the products, that drives the effects we hypothesize. In total, 47 out of the 178 participants indicated they bought one or more environmentally-friendly products; six participants did not answer this question. Second, participants who indicated they bought one or more environmentally-friendly products were asked to estimate the percentage of environmentally-friendly products out of their total purchase (*M* = 43.78%, *SD* = 27.64).

To examine how people's purchases were related to their self-image, participants then answered three statements reflecting one's environmental self-identity: “Behaving environmentally-friendly is an important part of who I am,” “I‘m the type of person that behaves environmentally-friendly,” and “I see myself as an environmentally-friendly person” (environmental self-identity; 1 = *totally disagree*, 7 = *totally agree*; Van der Werff et al., 2013). Mean scores of the three items were computed (α = 0.89, *M* = 4.23, *SD* = 1.16).

As a filler task, all participants additionally indicated why they made the purchases they did (“These products are better for the environment,” “Other people also bought these products,” “These products are healthy,” “These products are of good quality,” “I felt morally obliged to buy these products,” and “Other reason, namely …”). This list also included three items that were used to measure our moderator variable: volition of the purchase [“I wanted to buy these products,” “These products were the only products left in this category” (R) and “Somebody else asked me to buy these products” (R); 1 = *completely disagree*, 7 = *completely agree*]. The items that aimed to measure volition were not strongly interrelated (α = 0.20, *M*_*range*_ = 6.34 – 6.49, *SD*_*range*_ = 0.87 – 1.34). We therefore used the separate volition items as moderators in the analyses.

As our dependent variable, all participants indicated on a 7-point scale (1 = *completely disagree* to 7 = *completely agree*) to what extent purchasing the products they just paid for elicited each of six emotions: good, proud, cheerful (averaged to represent positive emotions; α = 0.75, *M* = 4.15, *SD* = 1.09), and as filler items: bad, guilty, and frustrated^1^. The questionnaire ended with demographics (age, gender, income, highest education level)^2^.

Participants were randomly assigned to either first answer the questions about how environmentally-friendly they perceived their purchases and themselves to be, respectively, or to first answer the questions about the emotions their purchases elicited. The questions about the reasons for purchase were always asked in-between these two blocks and the demographics were always asked last. The order of the questions did not affect any of our results.

### Results

Linear regression analysis shows that consumers who just bought environmentally-friendly products saw themselves as being more environmentally-friendly (*M* = 4.84) than consumers who did not buy environmentally-friendly products [*M* = 3.99; *B* = 0.86, *t*_(156)_ = 4.46, *p* < 0.001]. Furthermore, within the group of participants who indicated to have purchased environmentally-friendly products we found that the larger the share of environmentally-friendly products people bought, the more people saw themselves as an environmentally-friendly person [*B* = 0.01, *t*_(44)_ = 2.24, *p* < 0.05]. The extent to which the behavior was perceived as a voluntary choice^3^, however, was not found to affect the relationships between environmentally-friendly purchases and environmental self-identity, neither for purchasing vs. not purchasing environmental products (*B*_*interaction*_*yes*/*no*_ = −0.01, *p* = 0.95) nor for percentage of purchase (*B*_*interaction*_*percentage*_ = 0.00, *p* = 0.93). These results suggest that purchasing environmentally-friendly products is positively related to people's perception of themselves as environmentally-friendly, but volition did not significantly affect this relationship.

Next, we examined the relationship between environmental self-identity and the emotions elicited by the purchases. As expected, linear regression showed that people's environmental self-identity was related to how people felt about their purchases. The more they saw themselves as someone who acts environmentally-friendly, the better people felt about their purchases [*B* = 0.16, *t*_(156)_ = 2.16, *p* < 0.05]. Multiple linear regression showed that the relationship between environmental self-identity and positive emotions remained when controlled for environmental purchases (yes/no) [*B* = 0.18, *t*_(155)_ = 2.27, *p* < 0.05], but disappeared when controlled for percentage of purchase [*B* = 0.07, *t*_(43)_ = 0.45, *p* = 0.66].

Lastly, linear regression analysis did not show a total effect for environmental purchase: whether people just bought environmentally-friendly products was not found to be related to how positive they felt about their purchase [*B* = 0.01, *t*_(156)_ = 0.05, *p* = 0.96]. However, we did find a total effect for percentage of purchase: within the group that indicated to have purchased environmentally-friendly products, a larger share of environmentally-friendly products was related to feeling better about one's purchase [*B* = 0.01, *t*_(44)_ = 2.41, *p* < 0.05].

To test whether the self-signal ensuing from acting environmentally-friendly can account for the positive emotions this behavior elicits, we conducted a mediation analysis using bootstrapping (*N* = 1000; Preacher and Hayes, 2004). Our analysis showed that the indirect effect of environmentally-friendly purchase (yes/no) on positive emotions through environmental self-identity was significant (ab = 0.15, 95% CI [0.03,0.36]; see Table 1). These findings support the notion that consumers seem to feel better after purchasing environmentally-friendly products (vs. not buying environmentally-friendly products) because this behavior is related to a stronger environmental self-identity (i.e., people more strongly saw themselves as a person who acts pro-environmentally). No indirect effect through environmental self-identity was found for the relationship between percentage of environmentally-friendly purchase and positive emotions (ab = 0.0009, 95% CI [−0.002,0.005]; see Table 1)^4^.

**Table 1:** **Results of models testing the mediating effect of environmental self-identity on the relationship between environmentally-friendly purchase and positive emotions**.

	**Path A: effect purchase → self-identity**			**Path B: effect self-identity → positive emotions, controlled for purchase**			**Path C: effect purchase → positive emotions, controlled for self-identity**			**Path C': effect purchase → positive emotions**			**Bootstrap results for indirect effect**			
	***B***	***SE***	***t***	***B***	***SE***	***t***	***B***	***SE***	***t***	***B***	***SE***	***t***	**ab**	***SE***	**LL 95 CI**	**UL 95 CI**
Yes/No environmentally-friendly purchase	0.86^***^	0.19	4.46	0.18^*^	0.08	2.27	–0.14	0.20	–0.72	0.01	0.19	0.05	0.15	0.08	0.03	0.36
Percentage environmentally-friendly purchase	0.01^*^	0.006	2.24	0.07	0.16	0.45	0.01^*^	0.006	2.12	0.01^*^	0.006	2.41	0.0009	0.002	–0.002	0.005

ab, difference between the coefficient of (percentage of) environmentally-friendly purchase (X) in the analysis with (Path C) and the analysis without (Path C') environmental self-identity (M) as a covariate, LL 95 CI, Lower limit of 95% confidence interval; UL 95 CI, Upper limit of 95% confidence interval,

* p < 0.05,

*** p < 0.001(2-tailed).

### Discussion study 1

The results of Study 1 suggest that environmentally-friendly purchases are positively related to how environmentally-friendly people see themselves (i.e., their environmental self-identity), which in turn may account for the positive emotions these type of purchases elicits. This study only tested part of our reasoning, that is, we did not explicitly test yet whether environmentally-friendly behavior also boosts one's more overall positive self-image. As we reason in the introduction, environmentally-friendly behavior can be seen as a form of moral behavior. Thereby, seeing yourself as someone who acts environmentally-friendly could be interpreted as something positive: it would mean that you are someone who does good. In Study 2, we will test this reasoning further and look whether environmentally-friendly behavior can additionally boost people's more overall positive self-image, in turn eliciting positive emotions.

Although, Study 1 showed that environmentally-friendly purchases were related to a stronger environmental self-identity, and that a stronger environmental self-identity was related to more positive emotions about the purchase, causality could not be established because of the correlational design of the study. It could for instance also be possible that people who see themselves as being more environmentally-friendly want to act consistently with their identity, and purchase (a larger share of) environmentally-friendly products. We will elaborate on this point in the general discussion.

We hypothesized that behavior may more strongly signal something positive about you when the choice to engage in the behavior is made voluntarily, but this reasoning was not supported in Study 1. It would be premature, however, to conclude from the findings of Study 1 that volition does not influence the strength of the self-signal behavior sends. Since the average score on all volition items was high, a ceiling effect could explain this null-finding: people generally perceived their purchases to be their own choice. Furthermore, as the separate items measuring autonomy turned out to form an unreliable scale, we needed to rely on single items in our analyses. To gain more insight into the possible role of volition, we therefore manipulated rather than measured the extent to which the behavior is voluntary in Study 2.

## Study 2

### Method

We approached Dutch participants while they were waiting for or traveling by train, asking them whether they had time to complete a short survey. No incentives were provided for completion. In total, 159 people (85 female, 69 male, 5 unknown; *M*_*age*_ = 31.2 years, *SD*_*age*_ = 16.2; 56.8% of the sample had a monthly income of €500–€1000 or less; 66% of the sample had at least higher vocational education) agreed to complete the questionnaire that took approximately 5 min to fill out. In total, eight participants were excluded from the analyses because they had missing variables on at least one of the variables included in the analyses. The Ethical Committee Psychology of the University of Groningen approved Study 2 (approval number ppo-014-226). Informed consent was obtained from all participants.

Participants first indicated to what extent each of the following five statements about engagement in several environmentally-friendly behaviors were applicable to them (taking a shower that lasts <10 min, buying organic products, separating waste, using the bike for short distances and washing clothing on a low temperature; 1 = *not at all applicable to me*, 7 = *completely applicable to me*). As our independent variable, volition was manipulated as a between-subjects factor by introducing and framing these five behaviors as actions they decided to engage in out of their own volition (e.g., “I sometimes take a shower that lasts <10 min, even though I have enough time to stay in the shower for as long as I'd like”; volitional), or as behaviors participants engaged in out of situational constraints (e.g., “I sometimes take a shower that lasts <10 min because of time restraints”; non-volitional). By framing all behaviors as something people “sometimes” do, we made sure participants would find the behaviors applicable to themselves (*M* = 4.55, *SD* = 1.30; no difference in applicability found between volitional and non-volitional framing).

To examine to what extent the volition framing would affect the extent to which a positive self-image was elicited, participants then answered three statements: “The environmentally-friendly behaviors above … say something positive about who I am” “…indicate I'm a good person” “…show I'm someone who does the right thing” (1 = *completely disagree*, 7 = *completely agree*). Mean score on these items were computed (α = 0.85, *M* = 3.81, *SD* = 1.54).

As our dependent variable, participants then indicated to what extent the behaviors they just rated elicited each of 12 emotions on a 5-points scale (“When I engage in the environmentally-friendly behaviors above, it makes me feel…”; 1 = *not at all*, 3 = *moderately*, 5 = *very strongly*): good, satisfied, proud, happy, cheerful, inspired (averaged to represent positive emotions; α = 0.88, *M* = 2.92, *SD* = 0.90), and as filler items: frustrated, bad, uncomfortable, guilty, disappointed and unhappy^5^. The questionnaire ended with demographics (age, gender, income, highest education level).

### Results

As expected, linear regression analysis shows that participants felt the environmentally-friendly behaviors reflected more positively on who they are when these behaviors were voluntarily chosen (*M* = 4.13) than when these behaviors were not voluntarily chosen [*M* = 3.42; *B* = 0.70, *t*_(149)_ = 2.89, *p* < 0.01]. Furthermore, linear regression showed that the more positive the self-image behavior elicited was, the better people indicated feeling about acting accordingly [*B* = 0.28, *t*_(149)_ = 6.78, *p* < 0.001], also when controlled for volition [*B* = 0.30, *t*_(148)_ = 7.10, *p* < 0.001]. However, we did not find a total effect of voluntary (vs. non-voluntary) environmentally-friendly behavior on how people indicated feeling about acting accordingly [*B* = −0.04, *t*_(149)_ = −0.26, *p* = 0.80].

To test whether voluntary (vs. non-voluntary) engagement in environmentally-friendly behavior feels good because of the positive self-signal this behavior sends, we conducted a mediation analysis using bootstrapping (*N* = 1000; Preacher and Hayes, 2004). Our analysis showed that the indirect effect of voluntary (vs. non-voluntary) engagement in environmentally-friendly behavior on positive emotions through the positive self-image this behavior elicits was significant (ab = 0.21, 95% CI [0.07,0.36]; see Table 2). In other words, people seem to indicate feeling better about voluntary (vs. non-voluntary) environmentally-friendly behavior because particularly the former brings about a more positive self-image.

**Table 2:** **Results of model testing the mediating effect of positive self-image on the relationship between perceived environmentally-friendliness and positive emotions**.

**Path A: effect voluntary engagement → self-image**			**Path B: effect self-image à positive emotions, controlled for voluntary engagement**			**Path C: effect voluntary engagement à positive emotions, controlled for self-image**			**Path C': effect voluntary engagement à positive emotions**			**Bootstrap results for indirect effect**			
***B***	***SE***	***t***	***B***	***SE***	***t***	***B***	***SE***	***t***	***B***	***SE***	***t***	**ab**	***SE***	**LL 95 CI**	**UL 95 CI**
0.70^**^	0.24	2.89	0.30^***^	0.04	7.10	–0.25	0.13	–1.92	–0.04	0.15	–0.26	0.21	0.08	0.07	0.36

ab, difference between the coefficient of voluntary engagement (X) in the analysis with (Path C) and the analysis without (Path C') positive self-image (M) as a covariate, LL 95 CI, Lower limit of 95% confidence interval; UL 95 CI, Upper limit of 95% confidence interval,

** p < 0.01,

*** p < 0.001(2-tailed).

### Discussion study 2

The results of Study 2 suggest that reminding people of volitional (vs. non-volitional) environmentally-friendly behavior leads them to see themselves in a more positive light. In turn, this positive self-image may account for the positive emotions volitional environmentally-friendly behavior elicits. A limitation of Study 2 is that we did not include a manipulation check of volition. Therefore, we do not know for certain our manipulation indeed changed participants' sense of whether the environmentally-friendly behavior they were reminded of was volitional or not. Yet, the spoken responses we got from participants during the data collection give some indication our manipulation may have been successful: only in the situational constraint condition, participants felt the need to explain to us that they also engaged in these behaviors because they wanted to, and not only because of the situational constraints listed in the questionnaire. Nonetheless, as this provides only a tentative suggestion of the effectiveness of our manipulation, future research could include a manipulation check to be certain it is indeed volition that leads people to more strongly attribute behavior to themselves.

## General discussion

The goal of the current studies was to examine *why* engaging in environmentally-friendly behavior may contribute to individual well-being. More specifically, we examined the role one's self-image may play in explaining the relationship between environmentally-friendly behavior and positive emotions. We suggested acting environmentally-friendly itself may feel good because this behavior can signal something positive about who you are. Our findings suggest that engagement in environmentally-friendly behavior indeed is related to how people see themselves: the more environmentally-friendly their behavior, the more participants saw themselves as environmentally-friendly (Study 1). Furthermore, environmentally-friendly behavior was associated with a more general positive self-image, more strongly so when people were reminded of behavior they voluntarily engaged in, and not engaged in out of situational constraints (Study 2). In turn, the more environmentally-friendly (Study 1) and positively (Study 2) people saw themselves, the better they felt about acting environmentally-friendly. Together these results suggest that the positive self-signal that ensues from engaging in environmentally-friendly behavior may explain why acting this way can bring about a good feeling.

While we consistently found that how people saw themselves mediated the relationship between environmentally-friendly behavior and positive emotions, the evidence for the role of volition in strengthening this self-signal was mixed. While volition was not found to influence the relationship between environmentally-friendly behavior and environmental self-identity in Study 1, we did find that people saw themselves in a more general positive light after being reminded of environmentally-friendly behavior they engaged in voluntarily (vs. non-voluntarily). As discussed above, the null-findings in Study 1 may have been caused by a possible ceiling effect and the specific items used. Further, exploration of the role volition plays in the self-signal environmentally-friendly behavior sends and the positive emotions this behavior elicits could therefore provide fruitful new insights.

We also found mixed effects regarding a direct effect of environmentally-friendly behavior on positive emotions. Buying a larger share of environmentally-friendly products was linked to feeling more positive emotions, while buying environmentally-friendly products as such (yes/no) was not (Study 1). Furthermore, voluntary engagement in environmentally-friendly behavior was not found to make people feel better than engagement in environmentally-friendly behavior out of situational constraints (Study 2). When an indirect but no total effect is found, this may indicate that there are two opposite processes at work that suppress a total effect (Zhao et al., 2010). In the current studies that would mean that, while engagement in (voluntary) environmentally-friendly behavior may lead to a positive self-image and thereby feel good, the same engagement may also have negative side effects that decrease a positive feeling.

Following the reasoning above, future research could further investigate possible negative side effects that may reduce the positive feeling elicited by (voluntarily initiated) environmentally-friendly behavior. Firstly, processes outside of our theoretical framework may have influenced our results. As mentioned in the discussion of Study 2, some people in the non-voluntary condition felt the need to explain to us that they also voluntarily engage in the behaviors included in the questionnaire. This may indicate reactance could have played a role in the responses people gave. People in the non-voluntary condition may have felt the need to exaggerate the positive emotions elicited by the behaviors they just rated. This does raise the question, however, why they did show reactance on the questions regarding emotions, but not on the questions regarding positive self-image, where we did find the expected effect of our manipulation.

A theoretically interesting side effect to study would be that getting involved in environmental action also may involve becoming aware of the immensity of the problem we are facing. By getting familiar with the consequences our behavior has for nature and other people now and in the future, environmentally-friendly behavior becomes moral and good to engage in. At the same time, getting familiar with these consequences may lead people to realize many different actions are needed to have a substantial positive impact on the environment. This could have a disheartening effect, and the pursuit of unattainable goals leads to psychological distress (Emmons, 1986; Brunstein, 1993; Wrosch et al., 2003). Indeed, interviews with people who engage in environmental action show that they can feel angry or sad because of the bad state nature is in, the feeling they are not doing enough, and the idea that not enough people are doing their bit (Eigner, 2001). Buying environmentally-friendly products as such may thus make you see yourself as someone who acts environmentally-friendly, boosting your self-image, and therefore feeling good. However, at the same time it may make you realize much more action is necessary, therefore eliciting a bad feeling. Studying whether such simultaneous processes occur could provide fruitful insight in how to motivate people to engage in pro-environmental actions more often, while keeping them happy at the same time.

In addition to studying simultaneous processes that may work in opposite directions, another interesting direction would be to study simultaneous processes that all explain why environmentally-friendly behavior may feel good. Although the current study suggests that the positive self-signal that engaging in environmentally-friendly behavior sends may explain why acting this way can bring a good feeling, our findings do not imply this is the only reason such a positive relationship exist. An interesting additional explanation worth examining, is the sense of connectedness that environmentally-friendly behavior has been found to provide. As previous studies show, people who feel more connected to nature both act more environmentally-friendly (Mayer and Frantz, 2004; Nisbet et al., 2009) and feel better (Mayer et al., 2009; Ryan et al., 2010; Nisbet et al., 2011; Passmore and Howell, 2014). Whether a sense of connectedness can explain why environmentally-friendly behavior itself may feel good, however, is not clear yet.

Additional research on the causal relationship between the constructs of the current study could provide fruitful new insights as well. Study 2 suggests that the circumstances under which behavior occurs can influence the positivity of the self-image elicited. However, this study does not exclude that the relationship between behavior and self-image may also run in the other direction. In fact, it is likely that causality between environmentally-friendly behavior, self-image, and positive emotions runs in both directions. Research on environmentally-friendly behavior shows that previous behavior can influence the self-image people have, but that this self-image can also influence the behavior people engage in Van der Werff et al. (2014a). Furthermore, research on pro-social behavior shows that this type of behavior can make people feel good, but that people who feel good also act more pro-socially (Aknin et al., 2012). An important question to ask, therefore, is how a positive spiral of environmentally-friendly behavior, self-image and feeling good can be put in motion, and what the boundaries of this circular relationship are.

We started this paper by questioning whether environmentally-friendliness and feeling good are only linked more or less coincidentally, or whether engagement in environmentally-friendly behavior may also feel good in itself. The two studies described in this paper show that engagement in environmentally-friendly behavior can influence how people see themselves, which can explain how people feel about engagement in environmentally-friendly behavior. These results are a first indication that environmentally-friendly behavior may not just be a side-effect of doing the things that are known to contribute to a happy life, but that the behavior itself can also influence how people feel, through its effect on how they see themselves.

## Ethics statement

Ethical Committee Psychology of the University of Groningen. When approached participants were asked whether they wanted to participate in a short survey. Before completing the survey participants signed an informed consent form stating the duration of the study and explaining they could withdraw at any time, and that the data would be anonymous.

## Author contributions

LV, JB, and LS designed the studies in this article together. LV collected and analyzed the data. LV drafted the article and JB and LS engaged in several rounds of critical revision of the article.

## Funding

This research was part of a Ph.D. project funded by the European Commission 7th framework Programme for the project CReating Innovative Sustainability Pathways (CRISP).

### Conflict of interest statement

The authors declare that the research was conducted in the absence of any commercial or financial relationships that could be construed as a potential conflict of interest. The reviewer (AH) and the handling Editor declared their shared affiliation, and the handling Editor states that the process nevertheless met the standards of a fair and objective review.

## Footnotes

^1^Negative emotions were not found to be significantly related to any of our independent variables.

^2^Participants additionally indicated whether their purchase elicited a “feeling I did something fun or pleasant” and a “feeling I did something meaningful or valuable” (1 = completely disagree to 7 = completely agree). Results on these last two constructs showed the same pattern as the results reported for positive emotions.

^3^As the results for all three volition items were similar, we only report the results for the item “I wanted to buy these products” here. We found similar results when testing a moderated-mediation model using bootstrapping.

^4^This analysis was performed for the group of participants who indicated to have purchased one or more environmentally-friendly products (*N* = 47).

^5^Negative emotions were not found to be significantly related to any of our independent variables.
